# TabsPBP2, a Pheromone-Binding Protein Highly Expressed in Male Antennae of *Tuta absoluta*, Binds Sex Pheromones and Tomato Volatiles

**DOI:** 10.3390/biom15081152

**Published:** 2025-08-11

**Authors:** Cheng Qu, Jingxue Yan, Zuqing Yan, Ren Li, Yuqi Liu, Aoli Lin, Yuejun Fu, Chen Luo, Zhiwei Kang, Ran Wang

**Affiliations:** 1 Institute of Plant Protection, Beijing Academy of Agriculture and Forestry Sciences, Beijing 100097, China; qucheng@baaf.net.cn (C.Q.); yanjingxue@alu.sxu.edu.cn (J.Y.); yanzuqing@stumail.hbu.edu.cn (Z.Y.); liren@baafs.net.cn (R.L.); liuyuqi@stumail.hbu.edu.cn (Y.L.); aoli_lin@stumail.hbu.edu.cn (A.L.); 2 Beijing Key Laboratory of Environment Friendly Management on Fruit Diseases and Pests in North China, Beijing 100097, China; 3 Key Laboratory of Chemical Biology and Molecular Engineering of Ministry of Education, Institute of Biotechnology, Shanxi University, Taiyuan 030006, China; yjfu@sxu.edu.cn; 4 School of Life Science, Institutes of Life Science and Green Development, Hebei University, Baoding 071002, China; zwkang@hbu.edu.cn

**Keywords:** tomato leafminer, pheromone recognition mechanism, expression profile, fluorescence competition binding assay, molecular docking

## Abstract

The tomato leafminer (*Tuta absoluta*), a globally invasive pest, poses a major economic threat to tomato production. Although chemical control remains the primary management method, sustainable alternatives are urgently needed. Sex pheromone communication is critical for moth courtship and mating, with pheromone-binding proteins (PBPs) playing a key role in this process. In this study, we identified a PBP gene, *TabsPBP2*, from the *T. absoluta* transcriptome. Real-time quantitative PCR (RT-qPCR) revealed that *TabsPBP2* is highly expressed in the antennae, with a strong male-biased expression pattern. Ligand-binding assays demonstrated that TabsPBP2 has the highest affinity for the sex pheromones (3E, 8Z, 11Z)-tetradecatrienyl acetate (TDTA) and (3E, 8Z)-tetradecadienyl acetate (TDDA). It also demonstrated a moderate-to-strong binding affinity to several tomato volatiles, including 2-carene, myrcene, α-pinene, *cis*-3-hexen-l-ol, methyl salicylate, sabinene, and α-terpinene. Molecular docking suggested that hydrophobic interactions predominantly stabilize the TabsPBP2–ligand complexes, with PHE118, PHE12, LEU90, LEU68, and ALA73 identified as key interacting residues. Electroantennogram (EAG) and Y-tube olfactometer assays confirmed that TDTA and TDDA act as strong attractants for male *T. absoluta*. This study enhances our understanding of the pheromone recognition in *T. absoluta* and provides a foundation for developing novel, pheromone-based pest control strategies.

## 1. Introduction

The insect olfactory system enables the detection and discrimination of diverse chemical signals, such as sex pheromones and plant volatiles. This sensory capacity is essential for key behaviors, including the host location, food foraging, mate selection, habitat choice, and predator avoidance [1,2,3]. Odorant-binding proteins (OBPs) are the first to interact with odorants entering the sensillum, which transport these molecules through the sensillar lymph to ORs located on the membranes of olfactory sensory neurons [4]. Classified by the amino acid sequence homology, Lepidoptera OBPs comprise five subfamilies: pheromone-binding proteins (PBPs), general odorant-binding proteins type 1 and 2 (GOBP1 and GOBP2), and antennal-binding proteins type 1 and 2 (ABP1 and ABP2) [5]. The first PBP was identified in 1981 by Vogt et al. [6] in *Antheraea polyphemus* (Lepidoptera: Saturniidae) using labeled sex pheromones. Since then, PBPs have been characterized in various Lepidoptera families, including Noctuidae [7], Plutellidae [8], Pyralidae [9], and Arctiidae [10].

As small soluble proteins, PBPs contain six conserved cysteine residues that form three disulfide bridges, stabilizing their tertiary structure [11]. These proteins are typically abundant in male antennae, where they specifically bind and deliver female-emitted sex pheromones [12,13]. For example, *Spodoptera frugiperda* (Lepidoptera: Noctuidae) expresses four PBPs (*SfruPBP1-4*), among which SfruPBP1 exhibits a strong affinity for four sex pheromone components [14]. Some PBPs also interact with plant volatiles. In *Athetis lepigone* (Lepidoptera: Noctuidae), AlepPBP2 binds two pheromones and ten plant volatiles with high affinity [15,16]. Similarly, *Endoclita signifier* (Lepidoptera: Hepialidae) PBP3 (EsigPBP3) binds both the primary sex pheromone and seven major eucalyptus volatiles [17].

*Tuta absoluta* (Meyrick, 1917), a highly destructive pest originating from South America, has spread to more than 100 countries across Europe, the Middle East, and Africa [18,19,20,21,22]. It primarily targets tomato plants and other Solanaceae crops such as pepper, potato, tobacco, and eggplant, inflicting major economic losses [23,24]. Despite ongoing research into alternative management methods, chemical insecticides remain the primary control strategy [25,26]. However, over-reliance on chemical insecticides has resulted in resistance development and environmental concerns [27,28,29,30].

Pheromone-mediated control strategies, particularly mass trapping and mating disruption, have emerged as effective and environmentally friendly alternatives [31]. The female pheromone blend of *T. absoluta* (Lepidoptera: Gelechiidae) consists of (3E, 8Z, 11Z)-tetradecatrienyl acetate (TDTA) and (3E, 8Z)-tetradecadienyl acetate (TDDA) in a 90:10 ratio [32,33]. Attractants based on this blend have proven successful in monitoring and control [34,35]. To date, four PBPs (*TabsPBP1a*, *TabsPBP1b*, *TabsPBP1c*, and *TabsPBP3*) were identified in *T. absoluta* (Lepidoptera: Gelechiidae). Functional studies have been limited to TabsPBP3, which only exhibits a specific binding affinity for TDTA [36]. However, the specific PBPs that bind to TDDA in *T. absoluta* (Lepidoptera: Gelechiidae) remain unclear.

*TabsPBP2* was identified from the transcriptome of *T. absoluta* (Lepidoptera: Gelechiidae), showing male-biased and antenna-specific expression, suggesting a functional role in sex pheromone and plant volatile detection. Here, we investigated the binding properties of TabsPBP2 to sex pheromones and tomato volatiles. We also conducted three-dimensional protein modeling and molecular docking to predict the binding residues. Additionally, we validated the biological relevance of sex pheromones through electroantennography (EAG) and behavioral assays. Together, these results advance our understanding of TabsPBP2 and support the development of new, pheromone-based strategies for the sustainable management of *T. absoluta* (Lepidoptera: Gelechiidae).

## 2. Materials and Methods

### 2.1. Insect Rearing and Sample Collection

*T. absoluta* (Lepidoptera: Gelechiidae) was initially collected from tomato plants on a farm in Beijing, China, and subsequently reared in a growth chamber maintained at 25 ± 1 °C, 60% ± 5% relative humidity (RH), and a 16:8 h (L:D) photoperiod. Samples from various developmental stages—including eggs, first to fourth instar larvae, pupae, male adults, and female adults—were collected for developmental stage-specific expression analysis. For tissue-specific expression analysis, tissues from male adults (antennae, heads without antennae, thoraxes, abdomens, legs, and wings) were dissected. All samples were collected in triplicate and stored at −80 °C until use.

### 2.2. RNA Extraction and cDNA Synthesis

Total RNA extraction was carried out with TRIzol reagent (Invitrogen, Carlsbad, CA, USA), and first-strand cDNA was synthesized using a PrimeScript^TM^ RT Reagent Kit (Takara, Toyoko, Japan), both following the provided protocol.

### 2.3. Gene Cloning and Sequence Analysis

PCR was performed in a Bio-Rad DNA Engine Peltier Thermal Cycler (Bio-Rad, Hercules, CA, USA) using the following conditions: 94 °C (3 min); 35 cycles: 94 °C (40 s), 52 °C (50 s), and 72 °C (50 s); and final extension: 72 °C (10 min). Primer sequences are in Table S1.

Gel electrophoresis separation of PCR products preceded ligation into the pMD18-T vector (Takara, Toyoko, Japan) and transformed into *E. coli* DH5α competent cells (Takara, Toyoko, Japan). Tsingke (Beijing, China) performed plasmids sequencing. SignalP 5.0 (https://services.healthtech.dtu.dk/services/SignalP-5.0/ (accessed on 10 March 2023)) and ExPASy Proteomics Server (https://web.expasy.org/compute_pi/ (accessed on 20 March 2023)) were used for signal peptides prediction and molecular weight (MW)/isoelectric point (pI). Phylogenetic tree was generated based on the amino acid sequences of PBPs from *T. absoluta* (Lepidoptera: Gelechiidae) and other Lepidoptera species (*Grapholita funebrana* (Lepidoptera: Tortricidae): *GfunPBP1*: URZ86305.1, *GfunPBP1.2*: URZ86306.1, *GfunPBP2*: URZ86307.1, *GfunPBP3*: URZ86308.1; *Phauda flammans* (Lepidoptera: Phaudidae): *PflaPBP1*: QJZ31461.1, *PflaPBP2*: QJZ31460.1; *Helicoverpa armigera* (Lepidoptera: Noctuidae): *HarmPBP1*: AEB54585.1, *HarmPBP2*: AEB54583.1, *HarmPBP3*: AAO16091.1; *Spodoptera litura* (Lepidoptera: Noctuidae): *SlitPBP1*: AAY21255.1, *SlitPBP2*: AAZ22339.1, *SlitPBP3*: ACY78414.1; *Spodoptera exigua* (Lepidoptera: Noctuidae): *SexiPBP1*: AAS46620.1, *SexiPBP2*: AAS55551.2; *Plutella xylostella* (Lepidoptera: Plutellidae): *PxylPBP1*: BAG71422.1, *PxylPBP2*: AGH13203.1, *PxylPBP3*: ACI28451.1; *Grapholita molesta* (Lepidoptera: Tortricidae): *GmolPBP2*: AHZ89398.1, *GmolPBP3*: AHZ89399.1; *Scirpophaga excerptalis* (Lepidoptera: Pyralidae): *SexcPBP1*: AXF80669.1, *SexcPBP2*: AXF80670.1, *SexcPBP3*: AXF80671.1; *Agrotis ipsilon* (Lepidoptera: Noctuidae): *AipsPBP1*: AFM36756.1, *AipsPBP2*: AFM36757.1, *AipsPBP3*: AFM36758.1; *Maruca vitrata* (Lepidoptera: Crambidae): *MvitPBP1*: ANA06562.1, *MvitPBP2*: ANA06563.1, *MvitPBP3*: ANA06564.1; *Spodoptera frugiperda* (Lepidoptera: Noctuidae): *SfurPBP1*: QKX94922.1, *SfurPBP2*: QKX94923.1, *SfurPBP3*: QKX94924.1, *SfurPBP4*: QKX94925.1) in MEGA 5.0, applying the neighbor-joining method with 1000 bootstrap replicates.

### 2.4. Expression Profiling of PBPs

Gene expression was quantified by quantitative real-time PCR (RT-qPCR) with TB Green Premix Ex Taq^TM^ II (Takara, Japan) on a QuantStudio 7 Flex qRT-PCR System (Applied Biosystems, Waltham, MA, USA) using the following program: 95 °C (30 s); 40 × [95 °C (5 s), 60 °C (34 s)]. All reactions included triplicate biological and technical replicates. Primers design was performed via NCBI Primer-BLAST (https://www.ncbi.nlm.nih.gov/tools/primer-blast/index.cgi?LINK_LOC=BlastHome (accessed on 13 April 2023)), with EF1A and RPS13 using as internal reference genes [37]. The 2^−ΔΔCT^ method was used to analyze gene expression levels [38].

### 2.5. Recombinant Protein Expression and Purification

The *TabsPBP2* ORF (signal peptide excluded) was inserted into the pET30a vector via ClonExpressII One Step Cloning Kit (Vazyme, Nanjing, China) and expressed in *E. coli* BL21 (DE3) competent cells with 1.0 mM IPTG (37 °C, 4 h). Cells were harvested by centrifugation (12,100× *g*, 30 min, 4 °C) and lysed via sonication. TabsPBP2 was primarily expressed in inclusion bodies and denatured using 6 M guanidine hydrochloride. Solubilization and refolding of the recombinant protein were performed as described previously [39].

### 2.6. Competitive Fluorescence Binding Assay

Fluorescence binding assays were performed on a Cary Eclipse Fluorescence Spectrophotometer (Agilent Technologies, Palo Alto, CA, USA) to quantify TabsPBP2’s binding affinity for sex pheromones and tomato volatiles. Using n-phenyl-1-naphthylamine (1-NPN) as a fluorescent probe (1 mM in methanol), 2 μM TabsPBP2 (in 50 mM Tris-HCl, pH 7.4) were titrated with 1-NPN (2–16 µM). Fluorescence was excited at 337 nm with emission scanned from 350 and 500 nm. Ligand binding affinities were measured via competitive assays with 1-NPN (2 µM). Titration ranges: 0.5–4 μM (sex pheromones) and 2–50 μM (tomato volatiles). The binding constant (K_1-NPN_) for 1-NPN was calculated, followed by calculation of the dissociation constant (Ki) from IC_50_ values using the equation: Ki = [IC_50_]/(1 + [1-NPN]/K_1-NPN_), where [1-NPN] represents the free concentration of 1-NPN.

### 2.7. Modeling of TabsPBP2 and Ligand Docking

The 3D structure of TabsPBP2 was predicted by Swiss-Model [40]. The crystal structure of *Amyelois transitella* (Lepidoptera: Pyralidae) PBP1 (AtraPBP1, PDB ID: 4INX), which shares 60% sequence identity with TabsPBP2, was selected as the template. Structural optimization was carried out using the ff14SB force field with 7000 steps of energy minimization. Model validation using PROCHECK through Ramachandran plot confirmed that all amino acid residues fell within the allowed range for dihedral angles, indicating good model quality. Molecular docking was carried out using CB-Dock2 [41]. The predicted binding conformation was selected based on the highest docking score. Visualization of the docking results was performed by PyMOL (version 2.7).

### 2.8. Electrophysiological Recordings and Olfactometer Bioassay

Electroantennogram (EAG) recordings were performed to evaluate antennal responses of *T. absoluta* (Lepidoptera: Gelechiidae) to TDTA and TDDA, following the method described by Tu et al. (2024) [42]. Antennae from male adults were excised, with distal segments removed, and mounted between glass capillary electrodes. Each test chemical (1, 10, and 100 μg/μL, diluted in paraffin oil) was applied to a 1.0 cm × 3.0 cm filter paper strip, which was placed inside a glass Pasteur pipette to serve as a stimulus cartridge.

With a steady airflow of 10 mL/s, a stimulus controller (CS-55, Syntech, Kirchzarten, Germany) administered 0.5 s stimulation at 30 s intervals. EAG responses were recorded by EAG Pro software V2.02 (Syntech, Kirchzarten, Germany). A blank paraffin oil stimulus was applied before testing each compound. Six biological replicates (individual antennae) were analyzed per chemical, with net responses calculated by subtracting the blank control response.

Behavioral assays were conducted using a Y-tube olfactometer (Guangfahengtai, Beijing, China) to assess the responses of male *T. absoluta* (Lepidoptera: Gelechiidae) to TDTA and TDDA. The Y-tube olfactometer was constructed with a 10 cm main arm and 10 cm side arms forming 90° angles, with a 135° angle between the main arm (inner diameter: 1.5 cm) and the side arms (inner diameter: 1.5 cm). Air was filtered through activated carbon and divided into two equal streams at a flow rate of 250 mL/s.

Assays were performed under following conditions (25 ± 1 °C, 60% ± 5% RH, LED lighting). Each test solution (100 µg/µL in paraffin oil) was dispensed (10 μL) onto a 2 × 2 cm filter paper strip, which was placed at opposite sides inside an odor source bottle. Males were introduced individually into the base of the Y-tube and their behavior was observed for 5 min. An effective choice was recorded when the insect crossed the midpoint of either arm. The positions of odor sources were alternated after every five individuals to prevent positional bias. Sixty males were tested per compound.

### 2.9. Statistical Analysis

Data represent mean ± standard error (SE). Significant differences (*p* < 0.05) among groups were determined by one-way ANOVA with Tukey’s HSD test (SPSS, version 22.0). In the Y-tube olfactometer bioassay, *X*^2^ test was used to evaluate behavioral responses between sex pheromone and control.

## 3. Results

### 3.1. Sequence and Phylogenetic Analysis

**Figure 1:**
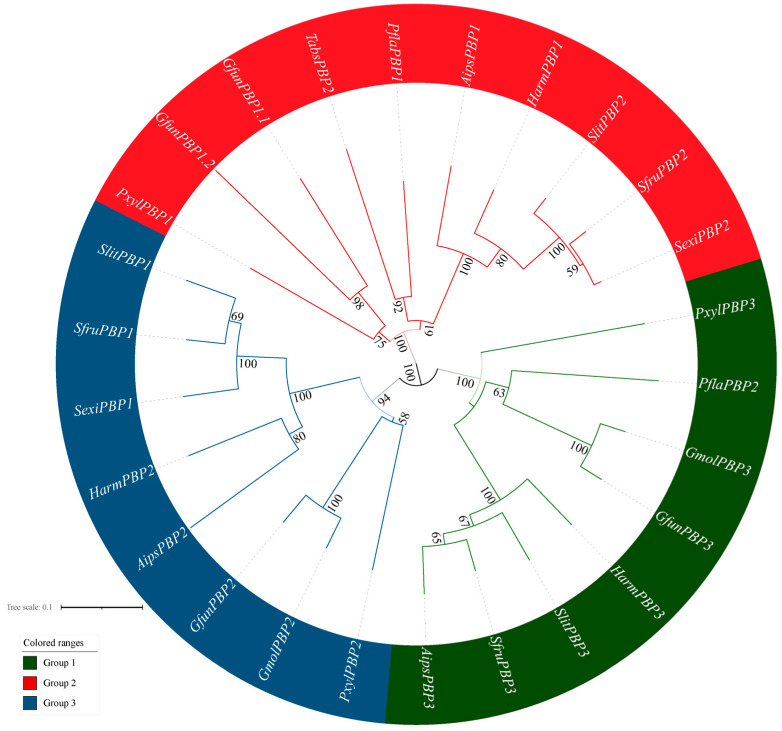
Phylogenetic tree of PBP amino acid sequences from *TabsPBP2* and other Lepidopteran insects.

The *TabsPBP2* nucleotide sequence was verified through molecular cloning and sequencing. Sequence analysis revealed that *TabsPBP2* encodes a full-length ORF comprising 162 amino acid residues, including a 20-residue N-terminal signal peptide. The predicted molecular weight and isoelectric point were 17.88 kDa and 5.14, respectively. Neighbor-joining (NJ) phylogenetic reconstruction indicated that *TabsPBP2* was grouped with PBP1 from *P. flammans* (Lepidoptera: Phaudidae) (Figure 1).

### 3.2. Expression Pattern of TabsPBP2

**Figure 2:**
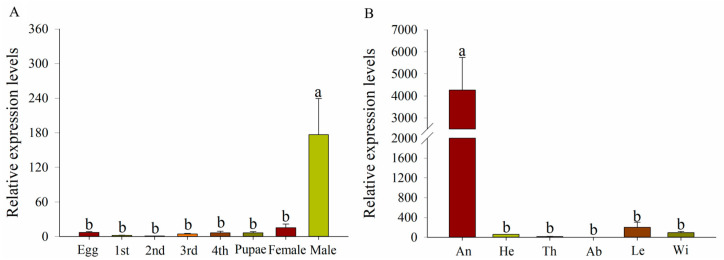
Expression patterns of *TabsPBP2* in various developmental stages (**A**). Expression patterns of *TabsPBP2* across various tissues in male adult (**B**) (An: antennae, He: head without antennae, Th: thorax, Ab: abdomen, Le: leg, Wi: wing). Different lowercase letters denote statistically significant differences (*p* < 0.05), as determined by one-way ANOVA followed by Tukey’s HSD test.

RT-qPCR analysis revealed stage-specific *TabsPBP2* expression peaking in male adults (Figure 2A), with tissue distribution analysis showing predominant expression in male adult antennae (Figure 2B).

### 3.3. Binding Characteristics of Recombinant TabsPBP2

**Figure 3:**
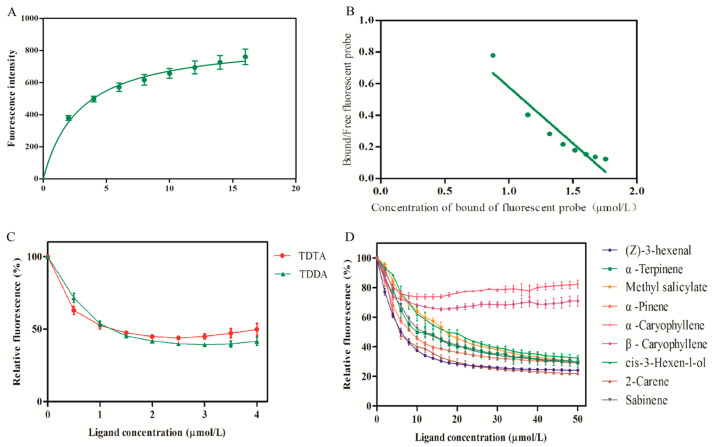
Binding curves and Scatchard plot for 1-NPN with TabsPBP2 (**A**,**B**). Competitive binding properties with sex pheromones (**C**). Competitive binding with tomato volatiles (**D**).

SDS-PAGE analysis confirmed the molecular weight and purity of recombinant TabsPBP2 (Figure S1). Competitive binding assays were conducted to evaluate the affinity of TabsPBP2 for sex pheromones and tomato volatiles. The dissociation constant (Ki) of TabsPBP2 for 1-NPN was determined to be 2.90 ± 0.48 μM. Fluorescence binding curves showed that TabsPBP2 bound strongly to two sex pheromones, TDTA (Ki = 1.39 ± 0.34 μM) and TDDA (Ki = 1.08 ± 0.08 μM). Among the nine tomato volatiles tested, high binding affinities were observed for 2-carene, myrcene, and α-pinene, with Ki values of 5.50 ± 0.92, 4.68 ± 0.66, and 7.48 ± 0.57 µM, respectively. Moderate binding affinity was found for *cis*-3-hexen-l-ol, methyl salicylate, sabinene, and α-terpinene, with Ki values of 13.65 ± 1.90, 13.02 ± 1.23, 11.41 ± 1.88, and 10.20 ± 3.60 µM, respectively (Figure 3 and Table S2).

### 3.4. Protein Modeling and Docking Analysis

**Figure 4:**
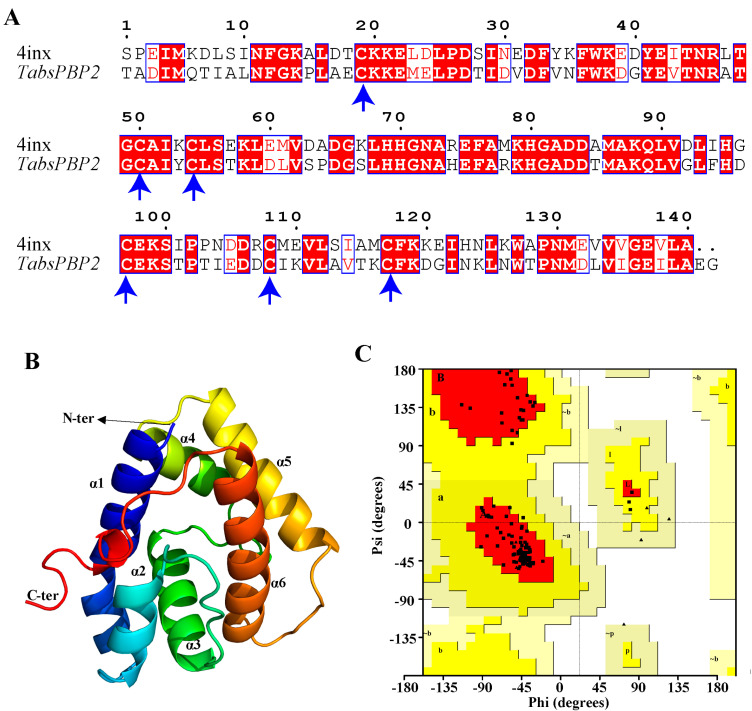
Sequence alignment between TabsPBP2 and AtraPBP1 (PDB ID: 4inx). The blue arrow identifies the conserved cysteine residue. Blue box denotes regions of sequence similarity between the two proteins (**A**). Predicted 3D structure of TabsPBP2 (**B**). Ramachandran plot of TabsPBP2 (**C**).

**Figure 5:**
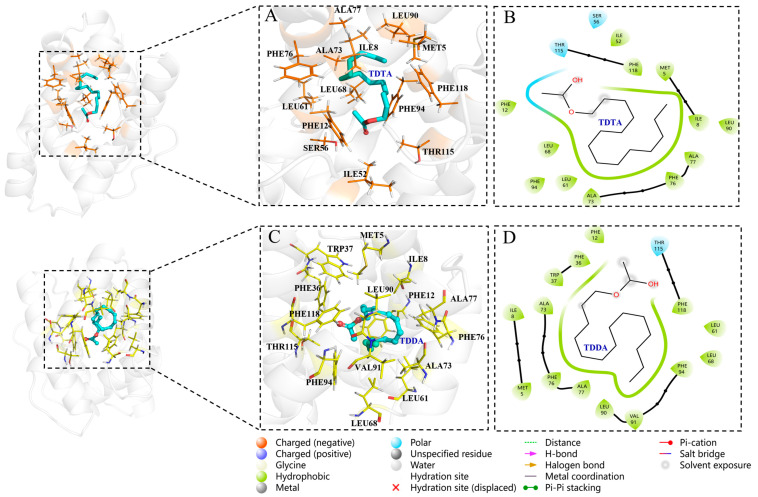
Binding mode diagrams and schematic illustrations of TabsPBP2 interactions with two sex pheromones: 3D structural mapping of the interaction between TDTA and amino acid residues of TabsPBP2 (**A**); 2D interaction diagram between TabsPBP2 binding sites and TDTA (**B**); 3D structural mapping of the interaction between TDDA and amino acid residues of TabsPBP2 (**C**); 2D interaction diagram between TabsPBP2 binding sites and TDDA (**D**).

**Figure 6:**
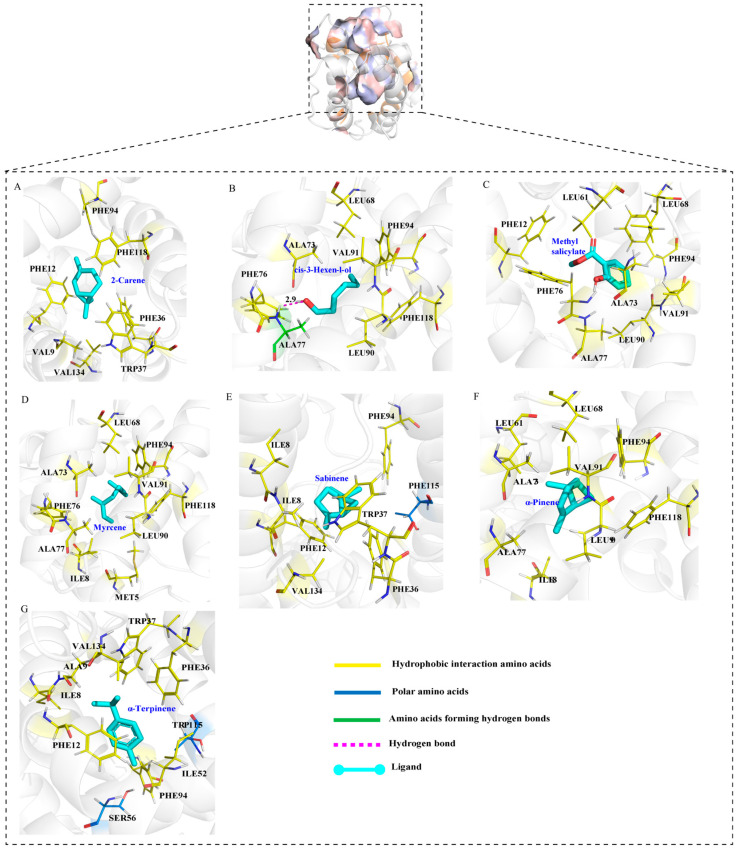
Binding mode diagrams and schematic illustrations of TabsPBP2 interactions with tomato volatiles: 3D representation of the interaction of 2-carene with TabsPBP2 residues (**A**); 3D representation of the interaction of *cis*-3-hexen-l-ol with TabsPBP2 residues (**B**); 3D representation of the interaction of methyl salicylate with TabsPBP2 residues (**C**); 3D representation of the interaction of myrcene with TabsPBP2 residues (**D**); 3D representation of the interaction of sabinene with TabsPBP2 residues (**E**); 3D representation of the interaction of α-pinene with TabsPBP2 residues (**F**); 3D representation of the interaction of α-Terpinene with TabsPBP2 residues (**G**).

The predicted 3D structure of TabsPBP2 exhibited six typical α-helices and an internal hydrophobic binding cavity, consistent with classic insect PBPs (Figure 4A,B). Structural validation using Procheck indicated that the model was reliable (Figure 4C). Molecular docking demonstrated that all ligands exhibited favorable binding to TabsPBP2, with binding energies ranging from −18.41 to −30.54 kJ/mol (Table S3). TDTA showed the strongest interaction energy (−30.54 kJ/mol). Hydrophobic interactions were identified as the primary stabilizing forces in the ligand–protein complexes, involving residues such as PHE118, PHE12, LEU90, LEU68, and ALA73. Polar interactions with SER56 and THR115 also contributed to binding (Figure 5). A hydrogen bond was observed between *cis*-3-hexen-l-ol and ALA77 (Figure 6).

### 3.5. EAG and Behavioral Responses of Male T. absoluta

**Figure 7:**
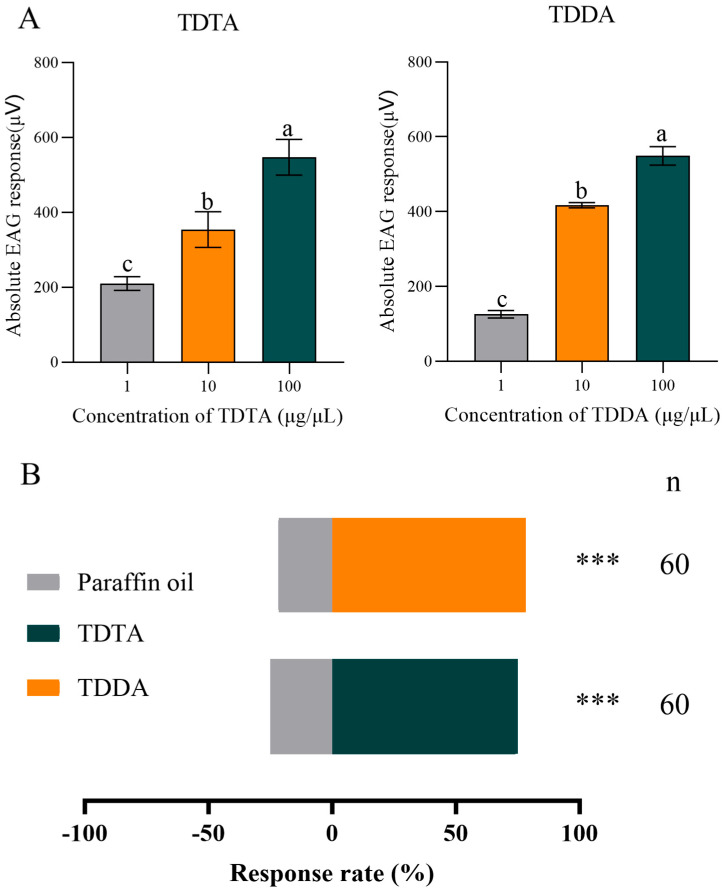
EAG responses of male *Tuta absoluta* antennae to TDTA and TDDA. (**A**) Different lowercase letters denote statistically significant differences (*p* < 0.05) as determined by one-way ANOVA followed by Tukey’s HSD test. Y-tube olfactometer behavioral responses to pheromones (**B**). Triple asterisks denote statistically highly significant differences (*p* < 0.001) by *X*^2^ test.

Electrophysiolographic (EAG) recordings were performed to quantify the response of male adults to TDTA and TDDA. The strongest responses were observed at 100 µg/µL, with mean EAG values of 547.33 ± 47.73 µV for TDTA and 549 ± 24.35 µV for TDDA (Figure 7A). In Y-tube olfactometer assays, 100 µg/µL of TDTA and TDDA significantly attracted male adults (Figure 7B).

## 4. Discussion

The sequence analysis detected six conserved cysteine residues in TabsPBP2, matching the signature pattern of insect OBPs. The phylogenetic analysis indicated a high degree of similarity between *TabsPBP2* and PBPs from other Lepidopteran species, consistent with the hypothesis for the monophyletic origin of moth PBPs followed by functional diversification through repeated gene duplication events [43]. In addition, about 27 OBPs genes were identified through the transcriptome sequencing of multiple tissues from adults of *T. absoluta* (unpublished data) and the number of TabsOBPs was less than that found in other Lepidopteran insects, such as 38 OBPs in *S. litura* (Lepidoptera: Noctuidae) [44], 39 OBPs in *P. xylostella* (Lepidoptera: Plutellidae) [45], and 40 OBPs in *Cydia pomonella* (Lepidoptera: Tortricidae) [46]. This discrepancy may be related to the insect species and sequencing methodology (the use of transcriptome sequencing instead of genome sequencing).

Olfactory-related genes expression exhibited distinct stage- and tissue-specific patterns, reflecting their specialized physiological functions [47,48]. In the present study, *TabsPBP2* was significantly overexpressed in male adults, particularly in the antennae, as confirmed by RT-qPCR. The male-biased expression pattern of *TabsPBP2* suggests its involvement in sexual communication, likely via the detection of sex pheromones. These findings are consistent with the antennal-specific expression of PBPs reported in other Lepidoptera species [49]. For example, Zhan et al. (2024) [50] reported the specific expression of *AaenPBP1* in the male antennae of *Agriphila aeneociliella* (Lepidoptera: Crambidae), while Si et al. (2022) [12] showed that PBP1-3 in *Orthaga achatina* (Lepidoptera: Pyralidae) exhibited male-biased expression in the antennae.

Fluorescent competitive binding assays revealed that TabsPBP2 possesses a high binding affinity for the two sex pheromones, TDTA and TDDA. Additionally, both EAG and Y-tube olfactometer experiments confirmed that male adults of *T. absoluta* (Lepidoptera: Phaudidae) were significantly attracted to these pheromone components. Similar findings have been documented in other Lepidopteran species. For instance, fluorescence binding assays revealed strong binding between MvitPBP1-3 from *M. vitrata* (Lepidoptera: Crambidae) and four sex pheromones [51], while SfurPBP1 and SfurPBP2 from *S. frugiperda* (Lepidoptera: Noctuidae) bound to three primary pheromone components [52].

In addition to pheromone recognition, PBPs have been implicated in the detection of plant volatiles. Strong binding affinity between TabsPBP2 and seven tomato volatiles, including myrcene, 2-carene, sabinene, α-terpinene, methyl salicylate, and *cis*-3-hexen-l-ol, were observed. This functional diversity highlights the critical role of TabsPBP2 in adult olfactory responses. Notably, methyl salicylate and β-myrcene have been shown to repel adult *T. absoluta* (Lepidoptera: Phaudidae) [53], and β-myrcene, (Ζ)-3-hexen-1-ol, and methyl salicylate were reported to elicit EAG responses in females [3], underscoring their importance in the species’ life cycle. Similar interactions have been observed in other Lepidoptera. For instance, binding assays demonstrated that EsigPBP3 from *Endoclita signifercan* (Lepidoptera: Hepialidae) binds key volatiles in eucalyptus leaves, including (−)-β-pinene, α-phellandrene, and eucalyptol, compounds known to mediate oviposition behavior [17]. Likewise, CpinPBP2 from *Conogethes pinicolalis* (Lepidoptera: Crambidae) was capable of binding both sex pheromones (E10-16: Ald and Z10-16: Ald) and multiple host plant volatiles (linalool, *cis*-3-hexen-1-ol, 1-octen-3-ol, limonene, and β-myrcene) [54].

Structural analysis revealed that TabsPBP2 adopts a conserved architecture, featuring six α-helices that form a hydrophobic ligand-binding pocket. This structural feature is consistent with previous reports on PxylPBP3 in *P. xylostella* (Lepidoptera: Plutellidae) [55] and HarmPBP1 in *H. armigera* (Lepidoptera: Noctuidae) [56], suggesting a conserved ligand-binding mechanism among PBPs. Molecular docking provided insights into both the key amino acid residues and the underlying binding mechanisms [57]. The binding efficiency of insect OBPs to ligands primarily depends on the interactions, including hydrogen bonds, van der Waals force, and hydrophobic interactions [58].

In agreement with the binding assays, molecular docking revealed low binding energies between TabsPBP2 and nine ligands, indicating strong affinities. Hydrophobic interactions constituted the primary stabilizing force within the binding pocket. Additionally, polar residues were found to contribute to the binding of TabsPBP2 to six ligands, and hydrogen bonding was also involved in the interaction with *cis*-3-hexen-1-ol. Several amino acid residues—including PHE118, PHE12, LEU90, LEU68, and ALA73—were involved in interactions with multiple ligands, indicating their potential importance in broad ligand recognition. Similar key residues, such as MET7, PHE35, PHE38, VAL10, ILE52, and PHE118, have been reported in EsigPBP2 from *Endoclita signifer* (Lepidoptera, Hepialidae) [17]. The functional roles of these key residues will be further examined by site-directed mutagenesis.

## 5. Conclusions

In conclusion, *TabsPBP2* was found to be highly expressed in the antennae of male *T. absoluta* adults. Fluorescence competitive binding analyses demonstrated a strong binding affinity for two sex pheromones and a moderate to strong binding affinity for tomato volatiles. Structural modeling and molecular docking analyses revealed the presence of six α-helices forming a stable hydrophobic ligand-binding pocket, with residues such as PHE118, PHE12, LEU90, LEU68, and ALA73 playing key roles in ligand binding. These findings suggest that TabsPBP2 is a key protein involved in the recognition of sex pheromones and host plant volatiles, providing valuable insights into the olfactory mechanisms of *T. absoluta*. Therefore, *TabsPBP2* may serve as a promising target for strategies aimed at disrupting chemical communication in this pest species.

## Data Availability

The original contributions presented in this study are included in the article/Supplementary Materials. Further inquiries can be directed to the corresponding authors.

## Supplementary Materials

The following supporting information can be downloaded at: https://www.mdpi.com/article/10.3390/biom15081152/s1, Figure S1: Expression and purification of *TabsPBP2*; Table S1: Primers used in this study; Table S2: Binding affinities of *TabsPBP2* for tested ligands; Table S3: Docking results for *TabsPBP2* with tested ligands.
