# Supplementary material for: TabsPBP2, a Pheromone-Binding Protein Highly Expressed in Male Antennae of Tuta absoluta, Binds Sex Pheromones and Tomato Volatiles
Source: Biomolecules. 2025 Aug 11;15(8):1152. doi: 10.3390/biom15081152 (PMC12384096; doi:10.3390/biom15081152)
Supplement: Supplementary file 1 [file biomolecules-15-01152-s001.zip › biomolecules-3759033-supplementary.pdf]

Table S1. Primers used in this study.

Primer name	Sequence (5'-3')	Use
TabsPBP2-ORF-F	ATGTTGAAGAAGGCGATTTTAATTT	Amplification
TabsPBP2-ORF-R	TTATCCTTCAGCTAAGATCTCGCCG	
TabsPBP2-Q-F	ATGGCTACGAGGTGACGAAC	qPCR
TabsPBP2-Q-R	GTCAGCGCCATGTTTTCTCG	
EF1α-F	AGTCTCCTCATACATCAAGAAG	
EF1α-R	CCTCCTTACGCTCAACAG	
RPS13-F	AGTTGCCCAAGTCAGATT	
RPS13-R	TTCCAAGTGCTTCCTCAT	
TabsPBP2-F	gccatggctgatatcggatccATGACGGCAGACATCATGCAAACT	Homologous recombination
TabsPBP2-R	ttgtcgacggagctcgaattcTTATCCTTCAGCTAAGATCTCGCC	

Table S2. Binding affinities of *TabsPBP2* for tested ligands.

Ligand	Source	CAS Number	Purity (%)	IC_{50} (μM)	K_i (μM)
(3E, 8Z, 11Z) tetradecatrienyl acetate	BIOBERRY	163041-94-9	85%	1.93 ± 0.50	1.39 ± 0.34
(3E, 8Z)-tetradecadienyl acetate	BIOBERRY	163041-87-0	92%	1.48 ± 0.11	1.08 ± 0.08
Myrcene	J&K Scientific	123-35-3	80%	6.22 ± 0.85	4.68 ± 0.66
2-Carene	Sigma	4497-92-1	97%	7.47 ± 1.20	5.50 ± 0.92
α-Pinene	Sigma	80-56-8	98%	9.98 ± 0.98	7.48 ± 0.57
Sabinene	MCE	3387-41-5	76.91%	13.62 ± 1.85	11.41 ± 1.88
α-Terpinene	Sigma	99-86-5	95%	14.59 ± 5.16	10.20 ± 3.60
Methyl salicylate	Sigma	119-36-8	99%	18.55 ± 1.38	13.02 ± 1.23
cis-3-Hexen-l-ol	J&K Scientific	928-96-1	98%	19.51 ± 2.39	13.65 ± 1.90
α-Caryophyllene	TCI	6753-98-6	93%	--	--
β- Caryophyllene	J&K Scientific	87-44-5	90%	--	--

Table S3. Docking results for *TabsPBP2* with tested ligands.

Ligand	Binding Energy (kJ/mol)	Hydrophobic Interactions	Polar Interactio ns	Hydrogen bond
(3E, 8Z, 11Z)-tetradecatrienyl acetate	-30.54	ILE52, ILE8, PHE118, PHE12, PHE94, PHE76, MET5, LEU90, LEU68, LEU61, ALA77, ALA73	SER 56, THR 115	
(3E, 8Z)-tetradecadienyl acetate	-27.20	ILE8, MET5, PHE12, PHE36, TRP37, ALA73, PHE76, ALA77, PHE118, LEU61, LEU68, PHE94, LEU90, VAL91	THR115	
Myrcene	-24.69	ALA73, LEU68, PHE118,		

		PHE76, ALA77, PHE94, VAL91, LEU90, MET5, ILE8		
2-Carene	-27.20	PHE94, PHE118, PHE12, VAL134, ALA9, TRP37, PHE36		
α-Pinene	-27.20	ALA73, LEU61, VAL91, PHE94, LEU68, LEU90, PHE118, ILE8, ALA77	SER 56, THR 115	
Sabinene	-25.94	PHE94, PHE12, ALA9, ILE8, PHE36, TRP37, VAL134	THR115	
α-Terpinene	-27.20	PHE94, SER56, ILE52, THR115, PHE12, VAL134, PHE36, TRP37, ALA9, ILE8		
Methyl salicylate	-22.59	LEU68, LEU61, ALA73, PHE12, PHE76, ALA77, LEU90, VAL91, PHE94		
cis-3-Hexen-l-ol	-18.41	LEU68, PHE94, PHE118, PHE76, VAL91, LEU90, ALA73		ALA77|N-H···O|OH|2.9|

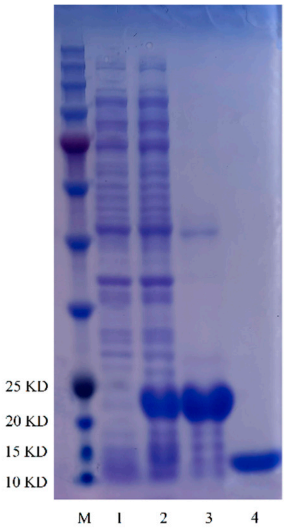

Figure S1 Expression and purification of TabsPBP2. M: protein marker; 1: TabsPBP2 protein without induced expression; 2: TabsPBP2 protein with induced expression; 3: recombinant TabsPBP2 protein after purification with His-tag; 4: TabsPBP2 protein without His-tag
